# Large retrospective study of artificial dura substitute in patients with traumatic brain injury undergo decompressive craniectomy

**DOI:** 10.1002/brb3.907

**Published:** 2018-03-25

**Authors:** Hongtao Sun, Hongda Wang, Yunfeng Diao, Yue Tu, Xiaohong Li, Wanyong Zhao, Jibin Ren, Sai Zhang

**Affiliations:** ^1^ Sixth Department of Neurosurgery, Brain Center Affiliated Hospital of Logistics University of People's Armed Police Force Tianjin China; ^2^ Division of Clinical Medicine Chongqing Medical University Chongqing China; ^3^ Brain Center Affiliated Hospital of Logistics University of People's Armed Police Force Tianjin China

**Keywords:** artificial dura material, bovine‐derived pericardium decompressive craniectomy, traumatic brain injury

## Abstract

**Background:**

Decompressive craniectomy is widely used for treating patients with traumatic brain injury (TBI). Usually patients have dura mater defect due to surgery or injury itself. The defective area may left open or repaired by artificial dura substitutes. A variety of artificial dura substitutes have been used for this purpose.

**Objective:**

This study aimed to evaluate bovine‐derived pericardium membrane as artificial dural material for patients with decompressive craniectomy.

**Methods:**

Totally 387 patients with severe TBI in our hospital were included in this study. Among them, 192 patients were treated with standard decompressive craniectomy without dura repair (control group). One hundred and ninety‐five TBI patients were treated with dura repair by artificial dura materials (ADM). Nonlyophilized bovine pericardium membranes were used as artificial dura material. The postoperative complications were compared in both groups, including infection, seizure, and cerebrospinal fluid (CSF) leakage.

**Results:**

Patients in control group have higher complication rates than patients in ADM group, including subcutaneous hematoma (13.02% in control vs. 4.01% in ADM group, *p* = .004), infection (12.5% in control vs. 5.64% in ADM group, *p* = .021), CSF leakage (13.02% in control vs. 5.13% in ADM group, *p* = .012), and seizure (10.42% in control vs. 3.08% in ADM group, *p* = .007). Patients in ADM group are only associated with higher incidence of foreign body reaction (6 of 195 patients in ADM vs. none from control group).

**Conclusion:**

Bovine‐derived pericardium membranes are successfully used as artificial dural substitutes for decompressive craniectomy. Patients with ADM have better clinical outcome than control group.

## 1 INTRODUCTION

Traumatic brain injury (TBI) is a complex injury with a broad spectrum of symptoms and disabilities, and it is the leading cause of death for individuals in our society between the ages of 1 and 45 (Rutland‐Brown, Langlois, Thomas, & Xi, 2006). Many survivors live with significant disabilities, resulting in major socioeconomic burden (Rutland‐Brown et al., 2006). Most of patients have mixed brain injuries, including brain concussion, subdural hematoma, and subarachnoid hemorrhage (Saatman et al., 2008). Patients with severe brain injury, based upon neurologic status defined by the Glasgow coma scale (GCS), usually need emergent surgical intervention, and decompressive craniectomy is a common procedure to release intracranial pressure (ICP) and open up brain cavity for further surgical treatment (Compagnone et al., 2005). Many patients may have significant dura mater defect due to TBI itself or by surgery. The defective dura area may be left open without repair or sealed with artificial dura substitutes (Huang, Lee, Chen, & Wang, 2011; Mundinger et al., 2016; Wang et al., 2015; Zhang, Yang, Jiang, & Zeng, 2010). Both procedures have been widely used and have distinctive advantages and disadvantages, for instance, patients without dura repair may have increased risk of CSF leakage and infection, while have better decompressive effect. In the meantime, dura repair with ADM may cause foreign body reaction and tissue capsule formation; however, it prevents CSF leakage and reduces the risk of intracranial infection (Cho & Kang, 2017; Zhang et al., 2017). So far there lacks of large clinical studies to compare clinical outcomes of both procedures in TBI patients.

Many materials have been studied for dura mater repair in patients with TBI, including silicon‐coated Dacron (Ongkiko, Keller, Mayfield, & Dunsker, 1984), reconstituted collagen foil (Pettorini et al., 2010), expanded polytetrafluoroethylene (ePTFE) porous material (Matsumoto et al., 2013), bioabsorbable polymers (Yamada et al., 2002), and nonabsorbable Neuro‐Patch (Huang et al., 2011). And some newly developed biomaterials show promising effect in animal models, such as poly (glycolide‐co‐lactide)/type I collagen/chitosan artificial (Bai, Wang, Yuan, Wang, & Wang, 2013), and cellulose knitted fabric (Suwanprateeb et al., 2016). Complications, such as capsule formation, hemorrhage, and extra‐axial hematoma formation, were reported caused by these materials (Huang et al., 2011; Matsumoto et al., 2013; Ongkiko et al., 1984). Recently, equine‐derived pericardium membrane was used a novel material for dura repair and showed significant therapeutic effect (Centonze, Agostini, Massaccesi, Toninelli, & Morabito, 2016). In this pilot study, none of the eight patients exhibited CSF leak, cerebral contusion, hemorrhage, or wound infection, as well as pseudomeningocele in a follow‐up examine (Centonze et al., 2016), indicating xenograft membrane is a good source for artificial dura substitute.

In this study, we used bovine‐derived pericardium membrane for dura repair in TBI patients and compared the clinical complications with or without dura repair after decompressive craniectomy.

## 2 PATIENTS AND METHODS

Criteria for patient selection: Totally 387 patients who had traumatic craniectomy from January 2011 to December 2013 in Affiliated Hospital of Logistics University of People's Armed Police Force were included in this study. A total of 192 patients from January 2011 to June 2012 in our hospital were treated with standard decompressive craniectomy without dura repair. After July 2012, dura mater repair was recommended for TBI patients as guideline changed, and 195 patients who received dura repair from July 2012 to December 2013 were included in this study. All patients had severe TBI based on the Glasgow coma scale (GCS ≤ 8). Most of them have complicated trauma, including subdural hematoma, brain contusion, intracranial hematoma, and subarachnoid hemorrhage.

Exclusion criteria: Patients with simple epidural hematoma, diffusive brain edema, and posterior cranial fossa trauma were excluded from this study. Patients with incomplete medical records and missing follow‐up information were excluded from this study.

### 2.1 Artificial dura substitutes

Biological dura mater patches were manufactured by Guanhao Biotech (Guangzhou, China). Briefly, bovine pericardium was harvested from 12‐month‐old 400–500 kg bovines (Chongqing Hengdu Food Development Co., Ltd. China) within 4 hr of sacrifice. The pericardium membranes were decellularized based on a previous study (Freytes, Martin, Velankar, Lee, & Badylak, 2008). The pericardium membranes were placed in aqueous peracetic acid (0.1% v/v) /ethanol (4% v/v) solutions for 2 hr. In the next step, pericardium membranes were washed by phosphate‐buffered saline (PBS, pH 7.4) and sterile deionized water, so as to remove the peracetic acid residue thoroughly. The decellularized pericardium membranes were fixed, and biological dura mater patches were fabricated. The biological dura mater patch was packaged and sterilized by γ‐irradiation at 25 kGy using a 60Co source. The dura patches were tested negative with prion protein as well as other pathogens, and it has met the criteria of China Food and Drug Administration regulation.

### 2.2 Surgical procedures

All the patients were under general anesthesia with airway intubation. The blood pressure, heart rate, and respiratory rate were closely monitored by anesthesiologists, and standard decompressive craniectomies were performed. The dura was cut in radial pattern or in arch shape, and damaged brain tissues and blood clot were removed. In ADM group, the dura was suspended along the cranial window, and defective areas were fixed by decellularized bovine‐derived pericardium membranes. The artificial membrane was decompressive sutured and sealed to the remaining dura mater. In control group, the dura was suspended along the cranial window, and the defective areas were left open without repair. In both groups, postoperative complications were closely monitored, including subcutaneous hematoma, foreign body reaction, intracranial infection, seizure, and fever. Information of surgery, such as duration of the operation, and blood loss, as well as degree of dura damage, was also collected for all patients.

### 2.3 Statistical analysis

SPSS software was used for statistical analysis. Student's *t* test was used to compare variables between control group and ADM group, and *p* < .05 is regarded as statistical significant. Logistic regression analyses were used to compare the odds ratio of postoperative complications in control and ADM group and adjusted with age and sex. *p* < .05 is considered as significant.

## 3 RESULTS

### 3.1 Demographic information of Patients

Totally 387 patients with severe TBI were included in this study, 192 patients were treated with standard decompressive craniectomy without dura repair (Control group), and 195 patients were treated with decompressive craniectomy followed artificial dura material repair (ADM group). A total of 150 male and 45 female patients were included in ADM group with average age of 43.83 ± 15.13 years old, and control group consisted of 130 male and 62 female patients with average age of 41.74 ± 15.67 years old (Table 1). There is no age and sex difference in both groups (Table 1). Patients from both groups have comparable severity of TBI based on the GCS scales obtained on initial examination (7.457.45 ± 2.76 in control vs. 7.53 ± 2.28 in ADM group, *p* = .379; Table 1). There is no difference in the types of TBI in both groups (*p* = .921; Table 1).

**Table 1:** Demographic information and type of trauma in control and AMD group

	Control group (*n* = 192)	ADM group (*n* = 195)	*p*
Ages, Mean ± *SD*	41.74 ± 15.69	43.83 ± 15.13	.183
Sex, *n*(%)			.033
Male	129 (67.19)	150 (76.92)	
Female	63 (32.81)	45 (23.08)	
GCS scale^a^, Mean ±* SD*	7.45 ± 2.76	7.53 ± 2.28	.379
Type of Trauma, *n*(%)			.921
Contusion	32 (16.67)	33 (16.92)	
Intracranial hematoma	11 (5.73)	9 (4.62)	
Subdural hematoma	143 (74.48)	145 (74.36)	
SAH	6 (3.13)	8 (4.10)	

SAH**,** subarachnoid hemorrhage; ADM, artificial dura materials; GCS, glasgow coma scale.

a GCS scales were obtained on initial examination.

### 3.2 Postoperative complications

Postoperative complications were closely monitored in all patients, including subcutaneous hematoma, seizure, intracranial infection, CSF leakage, and foreign body reaction. The results showed that patients in control group have more complications than patients in ADM group (Table 2); 13.02% patients in control group vs. 4.1% in ADM group had subcutaneous hematoma (OR = 3.349, 95% CI 1.460–7.678, *p* = .004 after adjustment), 12.5% patients in control group vs. 5.64% in ADM group had intracranial infection (OR = 2.432, 95% CI 1.144–5.170, *p* = .021 after adjustment), 13.02% patients in control group vs. 5.13% in ADM group had CSF leakage (OR = 2.689, 95% CI, 1.239–5.835, *p* = .012 after adjustment), and 10.42% in control group vs. 3.08% in ADM group developed seizure (OR = 3.705, 95% CI, 1.436–9.560, *p* = .007).

**Table 2:** Postoperative complications in control and ADM group

Complications	Control *n* = 192	ADM *n* = 195	Before adjustment		After adjustment	
			RR(95% CI)^a^	*p*	OR(95% CI)^b^	*p*
Hematoma	25 (13.02)	8 (4.10)	3.174 (1.468, 6.860)	.002	3.349 (1.460, 7.678)	.004
Foreign body reaction	0 (0.00)	6 (3.08)	—	.042	—	—
Intracranial infection	24 (12.50)	11 (5.64)	2.216 (1.117, 4.397)	.019	2.432 (1.144, 5.170)	.021
CSF leaking	25 (13.02)	10 (5.13)	2.539 (1.254, 5.143)	.007	2.689 (1.239, 5.835)	.012
Seizure	20 (10.42)	6 (3.08)	3.385 (1.390, 8.246)	.004	3.705 (1.436, 9.560)	.007

ADM, artificial dura materials; CSF, cerebrospinal fluid.

a Relative risk (RR) of complications in control and ADM group.

b Logistic regression analysis of odd ratio (OR) after adjustment of age, sex, GCS, and type of trauma. As there is no foreign body reaction in control group, RR and OR cannot be calculated in this situation.

Patients in both groups had comparable postoperative fever (4.41 ± 3.12 days in control group vs. 4.55 ± 3.2 days in ADM group, *p* = .771; Table 3). Some patients needed secondary cranioplasty due to large area of bone loss. Although the incidence of secondary surgery was comparable (38.54% in control vs. 42.56% in ADM group, *p* = .42), patients in ADM group had significant less duration of secondary cranioplasty (2.14 ± 0.36 hr in control vs. 1.61 ± 0.39 hr in ADM group, *p* < .001), less blood loss (205.81 ± 35.11 ml in control vs. 139.63 ± 37.46 in ADM group, *p* < .001), and slightly less postoperative infection after secondary cranioplasty (15.07% in control vs. 6.17% in ADM group, *p* = .071; Table 3).

**Table 3:** Comparison of postoperative fever and secondary surgery between control and ADM group

	Control group	ADM group	*p*
Fever (Day), Mean ±* SD*	4.41 ± 3.12	4.55 ± 3.2	.771
2nd surgery, *n*(%)	74 (38.54)	83 (42.56)	.420
Duration of 2nd surgery (h), Mean ± *SD*	2.14 ± 0.36	1.61 ± 0.39	<.001
Blood loss (ml), Mean ±* SD*	205.81 ± 35.11	139.63 ± 37.46	<.001
Infection after 2nd surgery	11 (15.07)	5 (6.17)	.071

ADM, artificial dura materials.

## 4 DISCUSSION

### 4.1 Goal of study

In this large retrospective study, we aimed to evaluate the therapeutic effect of bovine‐derived pericardium membrane as artificial dura material to repair dura defect of patients who had TBI.

### 4.2 Summary of results

One group of patients just receives standard decompressive craniectomy without dura repair (control group), and the other group receives dura repair after decompressive craniectomy (ADM group). The overall postoperative complications were compared in both groups, and the results showed that bovine‐derived pericardium membrane has overall better clinical outcomes than control group, and bovine‐derived pericardium membrane is good biomaterial for dura repair.

### 4.3 Comparison with other studies

Decompressive craniectomy has been widely used to treat patients with severe TBI. Usually, a part of skull and dura is removed in this procedure to reduce ICP, also open up cranial cavity to clear‐out blood clots and dead brain tissues. Although this procedure significantly releases ICP, its beneficial effect was controversial and its efficacy in TBI was uncertain (Citerio & Andrews, 2007; Ho, Honeybul, Lind, Gillett, & Litton, 2011; Maas, Stocchetti, & Bullock, 2008; Munch et al., 2000; Polin et al., 1997). Life‐threaten complications can occur after decompressive craniectomy, especially for elderly patients (De Bonis et al., 2011), such as hydrocephalus (De Bonis, Pompucci, Mangiola, Rigante, & Anile, 2010), interhemispheric subdural hygroma (De Bonis et al., 2013). However, decompressive craniectomy is not a risk factor for hemorrhagic contusions (Sturiale et al., 2012). A web‐based prognostic model has been developed to predict the outcome of decompressive craniectomy and evaluate the benefits and complications of decompressive craniectomy (Honeybul & Ho, 2014). However, there is a growing body of literature supporting the efficacy of decompressive craniectomy (Albanese et al., 2003; Mtaweh & Bell, 2015; Wang et al., 2015), including reducing ICP (Dam Hieu, Sizun, Person, & Besson, 1996), preventing brain edema (Burkert & Paver, 1988; Burkert & Plaumann, 1989; Guerra et al., 1999), and improving brain tissue oxygenation (Jaeger, Soehle, & Meixensberger, 2003).

In some cases, the defective dura will not be required after decompressive craniectomy, and patients usually have better decompressive effect, and shorter hospital stay and economically efficient. This procedure is still widely used especially in some underdeveloped area. More and more studies support to seal the defective dura with different biomaterials. There are three types of dura substitutes, based on their sources, allograft, xenograft, and biosynthesized materials. Allograft of dura is very limited due to the availability of the donor. Biosynthesized materials have been widely used in dura repair, including collagen film (Wang et al., 2013), silk fibroin (Kim et al., 2011), polyethylene glycol hydrogel (Jito, Nitta, & Nozaki, 2014; Osbun et al., 2012; Than, Baird, & Olivi, 2008), and ePTFE (Matsumoto et al., 2013; Wang et al., 2015; Yamagata, Goto, Oda, & Kikuchi, 1993), not only in TBI, also in other neurosurgeries, such as tumor dissection. However certain complications were associated with these biosynthesized materials. For instance, ePTFE dura materials cause inflammatory reaction and neoplastic tissue formation (El Majdoub et al., 2009).

Xenograft from other species has been used for this purpose. Xenogeneic dura mater progressively degraded over time, and no inflammatory cell response developed between the implant and the recipient brain parenchyma (Shi et al., 2009). And they are sufficiently biocompatible to allow epithelialization of its inner surface without adherence to brain tissue (Shi et al., 2009). Calf, porcine, ovine, equine, and bovine‐derived pericardium have been used as the source of dura materials (Centonze et al., 2016; Griessenauer et al., 2015; Parizek et al., 1989, 1996; Shi et al., 2009). Centonze et al. reported that eight patients received dura repair from equine‐derived pericardium, and no patients report CSF leak, cerebral contusion, hemorrhage, or wound infection. The 1‐month radiological follow‐up revealed no evidence of pseudomeningocele, wound breakdown, or meningitis (Centonze et al., 2016).

In addition to pericardium from different species, other tissues have been used to dura repair, such as porcine small intestinal submucosa (Bejjani & Zabramski, 2007). However, there is no study to compare which species is superior to the others in BTI treatment. Potentially, xerography from other species may cause zoonotic diseases, and most commonly used products are bovine growth hormone and dura mater grafts, that potentially will have prion contamination. Prions represent a group of proteins with a unique capacity to fold into different conformations. Pathogenic prions have been shown to cause lethal neurodegenerative diseases in humans and animals. These diseases are sometimes infectious and hence referred to as transmissible spongiform encephalopathies (Norrby, 2011). In the past over, 60% reported prion diseases caused by cadaveric dura mater transplantation were from Japan, and it is related to frequent use of Lyodura (Bonda et al., 2016). After improvement of manufacture, the incidence is significantly reduced (Bonda et al., 2016). In this study, we used decellularized bovine pericardium membranes which meet criteria of Chinese FDA, and de‐contamination of prion procedure has been performed. So far, no case of iatrogenic prion transmission has been reported with these products in China.

### 4.4 Limitations of current study

This is a retrospective study, and patients were included based on the information from medical records. Most of the patients did not have follow‐up examination. Therefore, we do not have long‐time safety data about bovine‐derived pericardium as ADM.

### 4.5 Ideas on future work

First, it is very important to address the safety and efficacy of bovine‐derived pericardium in a randomized clinical trial study, and follow‐up patients in a longer time period. Second, as calf, equine, and bovine‐derived pericardium were all used, it will be interesting to design studies to compare which one give the best clinical outcome.

## 5 CONCLUSION

In summary, our study demonstrated that bovine‐derived pericardium membranes are good artificial dural substitutes for decompressive craniectomy, and it associates less clinical complications than patients without dura repair.

## CONFLICT OF INTEREST

None declared.
